# Supplementary material for: Antidepressant prescriptions, discontinuation, depression and perinatal outcomes, including breastfeeding: A population cohort analysis
Source: PLoS One. 2019 Nov 18;14(11):e0225133. doi: 10.1371/journal.pone.0225133 (PMC6860440; doi:10.1371/journal.pone.0225133)
Supplement: S1 File — (DOCX) [file pone.0225133.s001.docx]

# S1 File. Read Codes for Depression

| Code Coding System Description | | |
| --- | --- | --- |
| Eu32. Read 5 | byte version 2 | Depressive episode |
| Eu320 Read 5 | byte version 2 | Mild depressive episode |
| Eu321 Read 5 | byte version 2 | Moderate depressive episode |
| Eu322 Read 5 | byte version 2 | Severe depressive episode without psychotic symptoms |
| Eu323 Read 5 | byte version 2 | Severe depressive episode with psychotic symptoms |
| Eu324 Read 5 | byte version 2 | Mild depression |
| Eu325 Read 5 | byte version 2 | Major depression, mild |
| Eu326 Read 5 | byte version 2 | Major depression, moderately severe |
| Eu327 Read 5 | byte version 2 | Major depression, severe without psychotic symptoms |
| Eu328 Read 5 | byte version 2 | Major depression, severe with psychotic symptoms |
| Eu329 Read 5 | byte version 2 | Single major depressive episode, severe, with psychosis, psychosis in remission |
| Eu32A Read 5 | byte version 2 | Recurrent major depressive episodes, severe, with psychosis, psychosis in remission |
| Eu32B Read 5 | byte version 2 | Antenatal depression |
| Eu32y Read 5 | byte version 2 | Other depressive episodes |
| Eu32z Read 5 | byte version 2 | Depressive episode, unspecified |
| E2B.. Read 5 | byte version 2 | Depressive disorder NEC |
| E2B0. Read 5 | byte version 2 | Postviral depression |
| E2B1. Read 5 | byte version 2 | Chronic depression |
| 1B17. Read 5 | byte version 2 | Depressed |
| 1B1U. Read 5 | byte version 2 | Symptoms of depression |
| 1BT.. Read 5 | byte version 2 | Depressed mood |
| 9H9.. Read 5 | byte version 2 | Mental health annual physical examination done |
| 9H90. Read 5 | byte version 2 | Depression annual review |
| 9H91. Read 5 | byte version 2 | Depression medication review |
| 9H92. Read 5 | byte version 2 | Depression interim review |
