# Supplementary material for: Antidepressant prescriptions, discontinuation, depression and perinatal outcomes, including breastfeeding: A population cohort analysis
Source: PLoS One. 2019 Nov 18;14(11):e0225133. doi: 10.1371/journal.pone.0225133 (PMC6860440; doi:10.1371/journal.pone.0225133)
Supplement: S2 File — (DOCX) [file pone.0225133.s002.docx]

## S2 File. Supplementary Tables

#### Table A1 Demographics details [ n=113316] Population, excluding infants with congenital anomalies.

|  | **>=1 SSRI [N06AB] in tri2-3** | | **>=1 high dose SSRI in t2-3** | | **>=1 antidepressant [N06A] in t2-3** | | **Depression diagnosis in GP records** | | **Unmedicated depression -no N06A in t2-3** | | **medicated depression N06A in t2-3** | | **Stopper >=1 SSRI in t1 but not t2-3** | | **stoppers>=1 antidepressant in t1 but not t2-3** | |
| --- | --- | --- | --- | --- | --- | --- | --- | --- | --- | --- | --- | --- | --- | --- | --- | --- |
|  | **exposed** | **not exposed** | **exposed** | **not exposed** | **exposed** | **not exposed** | **exposed** | **not exposed** | **exposed** | **not exposed** | **exposed** | **not exposed** | **exposed** | **not exposed** | **exposed** | **not exposed** |
|  | **N [%]** | **N [%]** | **N [%]** | **N [%]** | **N [%]** | **N [%]** | **N [%]** | **N [%]** | **N [%]** | **N [%]** | **N [%]** | **N [%]** | **N [%]** | **N [%]** | **N [%]** | **N [%]** |
| **Total** | **1820 [100]** | **111,496 [100]** | **608 [100]** | **112,708 [100]** | **2320 [100]** | **110,996 [100]** | **14,002 [100]** | **99,314 [100]** | **13,813 [100]** | **99,503 [100]** | **189 [100]** | **113,127 [100]** | **2514 [100]** | **110,802 [100]** | **4791 [100]** | **108,525 [100]** |
|  | | | | | | | | | | | | | | | | |
| **INSULIN_P1T1** | **11 [0.6]** | **356 [0.32]** | **<5** | **363-366 [0.32]** | **13 [0.56]** | **354 [0.32]** | **71-74 [0.51-0.53]** | **293-296 [0.30]** | **69-72 [0.50-0.52]** | **295-298 [0.3** **0** **]** | **<5** | **363-366 [0.32]** | **16 [0.64]** | **351 [0.32]** | **30 [0.63]** | **337 [0.31]** |
| **AEDS_P1T1** | **38 [2.09]** | **621 [0.56]** | **16 [2.63]** | **643 [0.57]** | **54 [2.33]** | **605 [0.55]** | **143-146 [1.02-1.04]** | **513-516 [0.52]** | **142-145 [1.03-1.05]** | **514-517 [0.52]** | **<5** | **655-658 [0.58]** | **28 [1.11]** | **631 [0.57]** | **104 [2.17]** | **555 [0.51]** |
| **ANTICOAGULANTS_P1T1** | **<5** | **44-47 [0.04]** | **<5** | **44-47 [0.04]** | **<5** | **44-47[0.04]** | **9 [0.06]** | **39 [0.04]** | **9 [0.07]** | **39 [0.04]** | **0** | **48 [0.04]** | **<5** | **44-47[0.04**  **]** | **<5** | **44-47[0.04]** |
|  | | | | | | | | | | | | | | | | |
| **BIRTH** | **N [%]** | **N [%]** | **N [%]** | **N [%]** | **N [%]** | **N [%]** | **N [%]** | **N [%]** | **N [%]** | **N [%]** | **N [%]** | **N [%]** | **N [%]** | **N [%]** | **N [%]** | **N [%]** |
| **singleton** | **1800 [98.9]** | **108,927 [97.7]** | **602 [99.01]** | **110,125 [97.71]** | **2292 [98.79]** | **108,435 [97.69]** | **13,698 [97.83]** | **97,029 [97.7]** | **13,513 [97.83]** | **97,214 [97.7]** | **185-188 [>97]** | **110,542 [97.71]** | **2443 [97.18]** | **108,284 [97.73]** | **4687 [97.83]** | **106,040 [97.71]** |
| **Twins and higher multipes** | **20 [1.11]** | **2569 [2.30]** | **6 [0.99]** | **2583 [2.29]** | **28 [1.21]** | **2561 [2.31]** | **303-306 [2.16-2.19]** | **2283-2286 [2.30]** | **296-299 [2.14-2.16]** | **2289-2292 [2.30]** | **<5** | **2585**  **-2588 [2.29]** | **71 [2.82]** | **2518 [2.27]** | **104 [2.17]** | **2485 [2.29]** |
|  | | | | | | | | | | | | | | | | |
| **INFANT OUTCOME** | **N [%]** | **N [%]** | **N [%]** | **N [%]** | **N [%]** | **N [%]** | **N [%]** | **N [%]** | **N [%]** | **N [%]** | **N [%]** | **N [%]** | **N [%]** | **N [%]** | **N [%]** | **N [%]** |
| **Live birth** | **1811 [99.51]** | **111,074 [99.62]** | **603-606 [>99]** | **112,280 [99.62]** | **2308 [99.48]** | **110,577 [99.62]** | **13,931 [99.49]** | **98,954 [99.64]** | **13,742 [99.49]** | **99,143 [99.64]** | **189 [100]** | **112,696 [99.62]** | **2498 [99.36]** | **110,387 [99.63]** | **4762 [99.39]** | **108,123 [99.63]** |
| **Stillborn** | **9 [0.49]** | **422 [0.38]** | **<5** | **427-430 [0.38**   **]** | **12 [0.52]** | **419 [0.38]** | **71 [0.51]** | **360 [0.36]** | **71 [0.51]** | **360 [0.36]** | **0** | **431 [0.38]** | **16 [0.64]** | **415 [0.37]** | **29 [0.61]** | **402 [0.37]** |
|  | | | | | | | | | | | | | | | | |
| **Substance misuse and/or heavy drinking** | **121 [6.65]** | **1674 [1.5]** | **45 [7.4]** | **1750 [1.55]** | **175 [7.54]** | **1620 [1.46]** | **697 [4.98]** | **1098 [1.11]** | **683 [4.94]** | **1112 [1.12]** | **14 [7.41]** | **1781 [1.57]** | **112 [4.46]** | **1683 [1.52]** | **298 [6.22]** | **1497 [1.38]** |
|  | | | | | | | | | | | | | | | | |
| **PARITY** | **N [%]** | **N [%]** | **N [%]** | **N [%]** | **N [%]** | **N [%]** | **N [%]** | **N [%]** | **N [%]** | **N [%]** | **N [%]** | **N [%]** | **N [%]** | **N [%]** | **N [%]** | **N [%]** |
| **primiparous** | **498 [27.36]** | **47,834 [42.9]** | **157 [25.82]** | **48,175 [42.74]** | **643 [27.72]** | **47,689 [42.96]** | **4464 [31.88]** | **43,868 [44.17]** | **4407 [31.9]** | **43,925 [44.14]** | **57 [30.16]** | **48,275 [42.67]** | **793 [31.54]** | **47,539 [42.9]** | **1430 [29.85]** | **46,902 [43.22]** |
| **multiparous** | **1322 [72.64]** | **63,662 [57.1]** | **451 [74.18]** | **64,533 [57.26]** | **1677 [72.28]** | **63,307 [57.04]** | **9538 [68.12]** | **55,446 [55.83]** | **9406 [68.1]** | **55,578 [55.86]** | **132 [69.84]** | **64,852 [57.33]** | **1721 [68.46]** | **63,263 [57.1]** | **3361 [70.15]** | **61,623 [56.78]** |
|  | | | | | | | | | | | | | | | | |
| **Year of Birth** | **N [%]** | **N [%]** | **N [%]** | **N [%]** | **N [%]** | **N [%]** | **N [%]** | **N [%]** | **N [%]** | **N [%]** | **N [%]** | **N [%]** | **N [%]** | **N [%]** | **N [%]** | **N [%]** |
| **2000** | **43 [2.36]** | **7981 [7.16]** | **10 [1.64]** | **8014 [7.11]** | **68 [2.93]** | **7956 [7.17]** | **456-459 [3.26-3.28]** | **7565-7568 [7.62]** | **453-456 [3.28-3.30]** | **7568-7571 [7.61]** | **<5** | **8020-8023 [7.09]** | **108 [4.3]** | **7916 [7.14]** | **188 [3.92]** | **7836 [7.22]** |
| **2001** | **63 [3.46]** | **8264 [7.41]** | **14 [2.3]** | **8313 [7.38]** | **97 [4.18]** | **8230 [7.41]** | **548-551[3.91-3.94]** | **7776-7779 [7.83]** | **543-546 [3.93-3.95]** | **7781-7784 [7.82]** | **5-8** | **8319-8322 [7.36]** | **156 [6.21]** | **8171 [7.37]** | **258 [5.39]** | **8069 [7.44]** |
| **2002** | **98 [5.38]** | **8522 [7.64]** | **25 [4.11]** | **8595 [7.63]** | **130 [5.6]** | **8490 [7.65]** | **628 [4.49]** | **7992 [8.05]** | **619 [4.48]** | **8001 [8.04]** | **9 [4.76]** | **8611 [7.61]** | **162 [6.44]** | **8458 [7.63]** | **295 [6.16]** | **8325 [7.67]** |
| **2003** | **146 [8.02]** | **9422 [8.45]** | **50 [8.22]** | **9518 [8.44]** | **199 [8.58]** | **9369 [8.44]** | **797 [5.69]** | **8771 [8.83]** | **789 [5.71]** | **8779 [8.82]** | **8 [4.23]** | **9560 [8.45]** | **226 [8.99]** | **9342 [8.43]** | **435 [9.08]** | **9133 [8.42]** |
| **2004** | **128 [7.03]** | **10,172 [9.12]** | **29 [4.77]** | **10271 [9.11]** | **176 [7.59]** | **10,124 [9.12]** | **1003 [7.16]** | **9297 [9.36]** | **991 [7.17]** | **9309 [9.36]** | **12 [6.35]** | **10,288 [9.09]** | **177 [7.04]** | **10,123 [9.14]** | **354 [7.39]** | **9946 [9.16]** |
| **2005** | **161 [8.85]** | **10,486 [9.4]** | **51 [8.39]** | **10596 [9.4]** | **228 [9.83]** | **10,419 [9.39]** | **1227 [8.76]** | **9420 [9.49]** | **1209 [8.75]** | **9438 [9.49]** | **18 [9.52]** | **10,629 [9.4]** | **217 [8.63]** | **10,430 [9.41]** | **457 [9.54]** | **10,190 [9.39]** |
| **2006** | **152 [8.35]** | **10,895 [9.77]** | **50 [8.22]** | **10,997 [9.76]** | **200 [8.62]** | **10,847 [9.77]** | **1396 [9.97]** | **9651 [9.72]** | **1377 [9.97]** | **9670 [9.72]** | **19 [10.05]** | **11,028 [9.75]** | **258 [10.26]** | **10,789 [9.74]** | **451 [9.41]** | **10,596 [9.76]** |
| **2007** | **208 [11.43]** | **11303 [10.14]** | **71 [11.68]** | **11440 [10.15]** | **251 [10.82]** | **11260 [10.14]** | **1693 [12.09]** | **9818 [9.89]** | **1673 [12.11]** | **9838 [9.89]** | **20 [10.58]** | **11491 [10.16]** | **279 [11.1]** | **11232 [10.14]** | **523 [10.92]** | **10988 [10.12]** |
| **2008** | **224 [12.31]** | **11473 [10.29]** | **82 [13.49]** | **11615 [10.31]** | **268 [11.55]** | **11429 [10.3]** | **1891 [13.51]** | **9806 [9.87]** | **1865 [13.5]** | **9832 [9.88]** | **26 [13.76]** | **11671 [10.32]** | **291 [11.58]** | **11406 [10.29]** | **545 [11.38]** | **11152 [10.28]** |
| **2009** | **279 [15.33]** | **11283 [10.12]** | **91 [14.97]** | **11471 [10.18]** | **324 [13.97]** | **11238 [10.12]** | **2055 [14.68]** | **9507 [9.57]** | **2022 [14.64]** | **9540 [9.59]** | **33 [17.46]** | **11529 [10.19]** | **321 [12.77]** | **11241 [10.15]** | **605 [12.63]** | **10957 [10.1]** |
| **2010** | **318 [17.47]** | **11695 [10.49]** | **135 [22.2]** | **11878 [10.54]** | **379 [16.34]** | **11634 [10.48]** | **2304 [16.45]** | **9709 [9.78]** | **2268 [16.42]** | **9745 [9.79]** | **36 [19.05]** | **11977 [10.59]** | **319 [12.69]** | **11694 [10.55]** | **680 [14.19]** | **11333 [10.44]** |
|  | | | | | | | | | | | | | | | | |
| **Maternal age, grouped** | **N [%]** | **N [%]** | **N [%]** | **N [%]** | **N [%]** | **N [%]** | **N [%]** | **N [%]** | **N [%]** | **N [%]** | **N [%]** | **N [%]** | **N [%]** | **N [%]** | **N [%]** | **N [%]** |
| **<20** | **86 [4.73]** | **9466 [8.49]** | **18 [2.96]** | **9534 [8.46]** | **111 [4.78]** | **9441 [8.51]** | **759 [5.42]** | **8793 [8.85]** | **752 [5.44]** | **8800 [8.84]** | **7 [3.7]** | **9545 [8.44]** | **180 [7.16]** | **9372 [8.46]** | **290 [6.05]** | **9262 [8.53]** |
| **20-24** | **373 [20.49]** | **23,594 [21.16]** | **115 [18.91]** | **23,852 [21.16]** | **465 [20.04]** | **23,502 [21.17]** | **3496 [24.97]** | **20,471 [20.61]** | **3451 [24.98]** | **20,516 [20.62]** | **45 [23.81]** | **23,922 [21.15]** | **688 [27.37]** | **23,279 [21.01]** | **1133 [23.65]** | **22,834 [21.04]** |
| **25-29** | **502 [27.58]** | **30,391 [27.26]** | **186 [30.59]** | **30,707 [27.24]** | **637 [27.46]** | **30,256 [27.26]** | **4041 [28.86]** | **26,852 [27.04]** | **3996 [28.93]** | **26,897 [27.03]** | **45 [23.81]** | **30,848 [27.27]** | **704 [28]** | **30,189 [27.25]** | **1315 [27.45]** | **29,578 [27.25]** |
| **30-34** | **510 [28.02]** | **29,822 [26.75]** | **180 [29.61]** | **30,152 [26.75]** | **655 [28.23]** | **29,677 [26.74]** | **3422 [24.44]** | **26,910 [27.1]** | **3369 [24.39]** | **26,963 [27.1]** | **53 [28.04]** | **30,279 [26.77]** | **551 [21.92]** | **29,781 [26.88]** | **1198 [25.01]** | **29,134 [26.85]** |
| **35-39** | **275 [15.11]** | **15,162 [13.6]** | **80 [13.16]** | **15,357 [13.63]** | **357 [15.39]** | **15,080 [13.59]** | **1878 [13.41]** | **13,559 [13.65]** | **1845 [13.36]** | **13,592 [13.66]** | **33 [17.46]** | **15,404 [13.62]** | **311 [12.37]** | **15,126 [13.65]** | **667 [13.92]** | **14,770 [13.61]** |
| **40-44** | **70-73 [nd]** | **2913 [2.61]** | **29 [4.77]** | **2957 [2.62]** | **91-94 [nd]** | **2893 [2.61]** | **389 [2.78]** | **2597 [2.61]** | **383 [2.77]** | **2603 [2.62]** | **6 [3.17]** | **2980 [2.63]** | **76-79 [nd]** | **2909 [2.63]** | **183 [3.82]** | **2803 [2.58]** |
| **>44** | **<5** | **137-140 [0.12-0.13]** | **0 [0]** | **141 [0.13]** | **<5** | **137-140 [0.12-0.13]** | **16 [0.11]** | **125 [0.13]** | **16 [0.12]** | **125 [0.13]** | **0 [0]** | **141 [0.12]** | **<5** | **137-140 [0.12-0.13]** | **5 [<0.01]** | **136 [0.13]** |
| **unknown** | **0** | **8 [0.01]** | **0** | **8 [0.01]** | **0** | **8 [0.01]** | **0** | **7 [0.01]** | **0** | **7 [0.01]** | **0** | **8 [0.01]** | **0** | **8 [0.01]** | **0** | **8 [0.01]** |
|  | | | | | | | | | | | | | | | | |
| **SMOKING** | **N [%]** | **N [%]** | **N [%]** | **N [%]** | **N [%]** | **N [%]** | **N [%]** | **N [%]** | **N [%]** | **N [%]** | **N [%]** | **N [%]** | **N [%]** | **N [%]** | **N [%]** | **N [%]** |
| **Non smoker** | **696 [38.24]** | **62,746 [56.28]** | **216 [35.53]** | **63,226 [56.1]** | **880 [37.93]** | **62,562 [56.36]** | **5786 [41.32]** | **57,656 [58.05]** | **5716 [41.38]** | **57,726 [58.01]** | **70 [37.04]** | **63,372 [56.02]** | **953 [37.91]** | **62,489 [56.4]** | **1785 [37.26]** | **61,657 [56.81]** |
| **Current smoker** | **874 [48.02]** | **32,655 [29.29]** | **312 [51.32]** | **33,217 [29.47]** | **1131 [48.75]** | **32,398 [29.19]** | **6028 [43.05]** | **27,501 [27.69]** | **5933 [42.95]** | **27,596 [27.73]** | **95 [50.26]** | **33,434 [29.55]** | **1157 [46.02]** | **32,372 [29.22]** | **2302 [48.05]** | **31,227 [28.77]** |
| **Total** | **1570 [86.26]** | **95,401 [85.56]** | **528 [86.84]** | **96,443 [85.57]** | **2011 [86.68]** | **94,960 [85.55]** | **11814 [84.37]** | **85,157 [85.75]** | **11649 [84.33]** | **85,322 [85.75]** | **165 [87.3]** | **96,806 [85.57]** | **2110 [83.93]** | **94,861 [85.61]** | **4087 [85.31]** | **92,884 [85.59]** |
| **Ex smoker** | **230 [12.64]** | **13,844 [12.42]** | **76-79 [nd]** | **13,998 [12.42]** | **284 [12.24]** | **13,790 [12.42]** | **2096 [14.97]** | **11,978 [12.06]** | **2072 [15]** | **12,002 [12.06]** | **24 [12.7]** | **14,050 [12.42]** | **370 [14.72]** | **13,704 [12.37]** | **644 [13.44]** | **13,430 [12.38]** |
| **unknown** | **20 [1.1]** | **2251 [2.02]** | **<5** | **2267-2270 [2.00]** | **25 [1.08]** | **2246 [2.02]** | **92 [0.66]** | **2179 [2.19]** | **92 [0.67]** | **2179 [2.19]** | **0** | **2271 [2.00]** | **34 [1.35]** | **2237 [2.02]** | **60 [1.25]** | **2211 [2.04]** |
|  | | | | | | | | | | | | | | | | |
| **BMI grouped** | **N [%]** | **N [%]** | **N [%]** | **N [%]** | **N [%]** | **N [%]** | **N [%]** | **N [%]** | **N [%]** | **N [%]** | **N [%]** | **N [%]** | **N [%]** | **N [%]** | **N [%]** | **N [%]** |
| **<20** | **152 [8.35]** | **9069 [8.13]** | **40 [6.58]** | **9181 [8.15]** | **202 [8.71]** | **9019 [8.13]** | **1415 [10.11]** | **7806 [7.86]** | **1395 [10.1]** | **7826 [7.87]** | **20 [10.58]** | **9201 [8.13]** | **230 [9.15]** | **8991 [8.11]** | **429 [8.95]** | **8792 [8.1]** |
| **20-24** | **499 [27.42]** | **33,080 [29.67]** | **173 [28.45]** | **33,406 [29.64]** | **617 [26.59]** | **32,962 [29.7]** | **4288 [30.62]** | **29,291 [29.49]** | **4232 [30.64]** | **29,347 [29.49]** | **56 [29.63]** | **33,523 [29.63]** | **697 [27.72]** | **32,882 [29.68]** | **1319 [27.53]** | **32,260 [29.73]** |
| **25-29** | **342 [18.79]** | **17,521 [15.71]** | **112 [18.42]** | **17,751 [15.75]** | **430 [18.53]** | **17,433 [15.71]** | **2606 [18.61]** | **15,257 [15.36]** | **2568 [18.59]** | **15,295 [15.37]** | **38 [20.11]** | **17,825 [15.76]** | **448 [17.82]** | **17,415 [15.72]** | **864 [18.03]** | **16,999 [15.66]** |
| **30-34** | **181 [9.95]** | **7407 [6.64]** | **63 [10.36]** | **7525 [6.68]** | **232 [10]** | **7356 [6.63]** | **1304 [9.31]** | **6284 [6.33]** | **1280 [9.27]** | **6308 [6.34]** | **24 [12.7]** | **7564 [6.69]** | **203 [8.07]** | **7385 [6.67]** | **419 [8.75]** | **7169 [6.61]** |
| **>34** | **126 [6.92]** | **4751 [4.26]** | **58 [9.54]** | **4819 [4.28]** | **176 [7.59]** | **4701 [4.24]** | **1026 [7.33]** | **3851 [3.88]** | **1009 [7.3]** | **3868 [3.89]** | **17 [8.99]** | **4860 [4.3]** | **194 [7.72]** | **4683 [4.23]** | **373 [7.79]** | **4504 [4.15]** |
| **unknown** | **520 [28.57]** | **39,668 [35.58]** | **162 [26.64]** | **40,026 [35.51]** | **663 [28.58]** | **39,525 [35.61]** | **3363 [24.02]** | **36,825 [37.08]** | **3329 [24.1]** | **36,859 [37.04]** | **34 [17.99]** | **40,154 [35.49]** | **742 [29.51]** | **39,446 [35.6]** | **1387 [28.95]** | **38,801 [35.75]** |
|  | | | | | | | | | | | | | | | | |
| **TOWNSEND_FIFTH** | **N [%]** | **N [%]** | **N [%]** | **N [%]** | **N [%]** | **N [%]** | **N [%]** | **N [%]** | **N [%]** | **N [%]** | **N [%]** | **N [%]** | **N [%]** | **N [%]** | **N [%]** | **N [%]** |
| **Townsend fifth** | **229 [12.58]** | **19,499 [17.49]** | **81-84 []** | **19,645 [17.43]** | **277 [11.94]** | **19,451 [17.52]** | **1830 [13.07]** | **17,898 [18.02]** | **1799 [13.02]** | **17,929 [18.02]** | **29-32 []** | **19,697 [17.41]** | **280-283 []** | **19,447 [17.55]** | **537 [11.21]** | **19,191 [17.68]** |
| **1 [least deprived]** |  |  |  |  |  |  |  |  |  |  |  |  |  |  |  |  |
| **2** | **331 [18.19]** | **21,318 [19.12]** | **100 [16.45]** | **21,549 [19.12]** | **395 [17.03]** | **21,254 [19.15]** | **2171 [15.5]** | **19,478 [19.61]** | **2137 [15.47]** | **19,512 [19.61]** | **34 [17.99]** | **21,615 [19.11]** | **384 [15.27]** | **21,265 [19.19]** | **763 [15.93]** | **20,886 [19.25]** |
| **3** | **334 [18.35]** | **21,747 [19.5]** | **115 [18.91]** | **21,966 [19.49]** | **436 [18.79]** | **21,645 [19.5]** | **2587 [18.48]** | **19,494 [19.63]** | **2562 [18.55]** | **19,519 [19.62]** | **25 [13.23]** | **22,056 [19.5]** | **498 [19.81]** | **21,583 [19.48]** | **934 [19.49]** | **21,147 [19.49]** |
| **4** | **402 [22.09]** | **23,824 [21.37]** | **136 [22.37]** | **24,090 [21.37]** | **520 [22.41]** | **23,706 [21.36]** | **3336 [23.83]** | **20,890 [21.03]** | **3285 [23.78]** | **20,941 [21.05]** | **51 [26.98]** | **24,175 [21.37]** | **586 [23.31]** | **23,640 [21.34]** | **1080 [22.54]** | **23,146 [21.33]** |
| **5 [most deprived]** | **519 [28.52]** | **24,660 [22.12]** | **172 [28.29]** | **25,007 [22.19]** | **686 [29.57]** | **24,493 [22.07]** | **4052 [28.94]** | **21,127 [21.27]** | **4006 [29]** | **21,173 [21.28]** | **46 [24.34]** | **25,133 [22.22]** | **762 [30.31]** | **24,417 [22.04]** | **1471 [30.7]** | **23,708 [21.85]** |
| **Unkown** | **5 [0.27]** | **448 [0.40]** | **<5** | **449-452 [0.40]** | **6 [0.26]** | **447 [0.4]** | **26 [0.19]** | **427 [0.43]** | **24 [0.17]** | **429 [0.43]** | **<5** | **449-452 [0.40]** | **<5** | **449-452 [0.41]** | **6 [0.13]** | **447 [0.40]** |
|  | | | | | | | | | | | | | | | | |
|  | **Mean [sd]** | **Mean [sd]** | **Mean [sd]** | **Mean [sd]** | **Mean [sd]** | **Mean [sd]** | **Mean [sd]** | **Mean [sd]** | **Mean [sd]** | **Mean [sd]** | **Mean [sd]** | **Mean [sd]** | **Mean [sd]** | **Mean [sd]** | **Mean [sd]** | **Mean [sd]** |
| **Time on database before LMP [years]** | **8.13 [5.28]** | **7.62 [4.93]** | **8.21 [5.34]** | **7.62 [4.94]** | **8.06 [5.22]** | **7.62 [4.93]** | **9.62 [4.94]** | **7.35 [4.87]** | **9.62 [4.94]** | **7.35 [4.87]** | **9.61 [4.63]** | **7.62 [4.94]** | **8.09 [5.06]** | **7.62 [4.94]** | **8.03 [5.11]** | **7.61 [4.93]** |
| **MATERNAL AGE** | **28.97 [5.91]** | **28.21 [6.07]** | **29.26 [5.62]** | **28.22 [6.07]** | **29.06 [5.91]** | **28.21 [6.07]** | **28.21 [5.87]** | **28.22 [6.09]** | **28.2 [5.87]** | **28.23 [6.09]** | **29 [5.95]** | **28.22 [6.06]** | **27.71 [6.05]** | **28.23 [6.06]** | **28.44 [6.06]** | **28.21 [6.06]** |
| **BMI** | **25.88 [6.48]** | **24.81 [5.79]** | **26.61 [6.92]** | **24.82 [5.79]** | **26 [6.58]** | **24.81 [5.78]** | **25.57 [6.49]** | **24.71 [5.67]** | **25.56 [6.48]** | **24.71 [5.67]** | **26.28 [7.07]** | **24.83 [5.8]** | **25.83 [6.76]** | **24.81 [5.77]** | **25.89 [6.7]** | **24.78 [5.75]** |
| **TOWNSEND SCORE** | **0.96 [3.33]** | **0.29 [3.17]** | **1.03 [3.4]** | **0.29 [3.17]** | **1.05 [3.29]** | **0.28 [3.17]** | **0.93 [3.23]** | **0.21 [3.15]** | **0.93 [3.23]** | **0.21 [3.15]** | **0.71 [3.32]** | **0.3 [3.17]** | **1.08 [3.23]** | **0.28 [3.17]** | **1.1 [3.25]** | **0.26 [3.16]** |
| **TOWNSEND FIFTH** | **3.36 [1.39]** | **3.12 [1.41]** | **3.35 [1.4]** | **3.12 [1.41]** | **3.41 [1.38]** | **3.11 [1.41]** | **3.4 [1.38]** | **3.08 [1.41]** | **3.4 [1.38]** | **3.08 [1.41]** | **3.25 [1.43]** | **3.12 [1.41]** | **3.46 [1.35]** | **3.11 [1.41]** | **3.46 [1.36]** | **3.1 [1.41]** |

Notes:

Exclusions: all congenital anomalies and TOPFAs. This information is published elsewhere.

Some numbers have been blurred to avoid revealing numbers <5 in other cells.

BMI is body mass index, LMP is last menstrual period, t2-t3 represents trimesters 2 and 3, P1_t1 represents the quarter before pregnancy and trimester 1.

Townsend scores and ranks, see note to Table 2a, reference 89

#### Table A2 Demographics Details of Subjects Included in the Analysis (n=107573)

|  | **Population (exclusions listed below)** | **>=1 SSRI (N06AB) in tri2-3** | **>=1 high dose SSRI in t2-3** | **>=1 antidepressant (N06A) in t2-3** | **Depression diagnosis in GP records** | **Unmedicated depression -no N06A in t2-3** | **Medicated depression N06A in t2-3** | **Stopper >=1 SSRI in t1 but not t2-3** | **stoppers>=1 antidepressant in t1 but not t2-3** |
| --- | --- | --- | --- | --- | --- | --- | --- | --- | --- |
| **Number of infants** | n(%) | n(%) | n(%) | n(%) | n(%) | n(%) | n(%) | n(%) | n(%) |
| **Total** | 107,573 (100) | 1625 (100) | 538 (100) | 2043 (100) | 12,748 (100) | 11,700 (100) | 1048 (100) | 2285 (100) | 4252 (100) |
| **Parity:** |  |  |  |  |  |  |  |  |  |
| Primiparious | 45,483 (42.3) | 448 (27.6) | 140 (26) | 573 (28) | 3964 (31.1) | 3671 (31.4) | 293 (28.0) | 682 (29.8) | 1243 (29.2) |
| Multiparious | 62,090 (57.7) | 1177 (72.4) | 398 (74) | 1470 (72) | 8784 (68.9) | 8029 (68.6) | 755 (72.0) | 1603 (70.2) | 3009 (70.8) |
|  |  |  |  |  |  |  |  |  |  |
| **Mean age** at pregnancy end [years: (SD)] | 28.20 [6.06] | 29.03 (5.9) | 29.32 (5.6) | 29.11 (5.9) | 28.25 (5.8) | 28.15 [5.9] | 29.31 [5.7] | 27.76 (6.0) | 28.46 (6.06) |
| <20 | 9114 (8.5) | 75(4.6) | 14 (2.6) | 93 (4.6) | 679 (5.3) | 650 (5.6) | 29 (2.8) | 155 (6.8) | 247 (5.8) |
| 20-24 | 22,808 (21.2) | 331 (20.4) | 101 (18.8) | 410 (20.1) | 3138 (24.6) | 2933 (25.1) | 205 (19.6) | 628 (27.5) | 1015 (23.9) |
| 25-29 | 29,415 (27.3) | 446 (27.4) | 167 (31) | 560 (27.4) | 3722 (29.2) | 3408 (29.1) | 314 (30.0) | 639 (28) | 1172 (27.6) |
| 30-34 | 28,716 (26.7) | 455 (28.0) | 158 (29.4) | 575 (28.1) | 3117 (24.5) | 2822 (24.1) | 295 (28.1) | 503 (22) | 1048 (24.6) |
| 35-39 | 14,577 (13.6) | 249 (15.3) | 70 (13) | 316 (15.5) | 1713 (13.4) | 1551 (13.3) | 162 (15.5) | 283 (12.4) | 594 (14) |
| 40-44 | 2824 (2.6) | 69 (4.3) | 28 (5.2) | 89 (4.4) | 367 (2.9) | 325 (2.8) | 43 (4.1) | 77 (3.3) | 171 (4) |
| >44 | 111 (0.1) | § | § | § | 11 (0.1) | 10 (0.1) | § | § | 5 (0.1) |
| unknown | 8 (0.0) | 0 | 0 | 0 | 1 (0) | 1 (0) | 0 | 0 | 0 |
| **Smoking status** |  |  |  |  |  |  |  |  |  |
| non-smoker | 60,901 (56.6) | 647 (39.8) | 204 (37.9) | 816 (39.9) | 5455 (42.8) | 5054 (43.2) | 401 (38.3) | 899 (39.3) | 1667 (39.2) |
| current smoker | 31,069 (28.9) | 744 (45.8) | 256 (47.6) | 941 (46.1) | 5268 (41.3) | 4775 (40.8) | 493 (47.0) | 1014 (44.4) | 1939 (45.6) |
| ex-smoker | 13,428 (12.5) | 217 (13.4) | 74 (13.8) | 265 (13) | 1946 (15.3) | 1801 (15.4) | 145 (13.9) | 342 (15) | 591 (13.9) |
| unknown | 2175 (2.0) | 17 (1) | 4 (0.7) | 21 (1) | 79 (0.6) | 70 (0.6) | 9 (0.9) | 30 (1.3) | 55 (1.2) |
| **Socioeconomic status** |  |  |  |  |  |  |  |  |  |
| Wales Townsend fifth = 1 (least deprived) | 18,910 (17.6) | 216 (13.3) | 79 (14.7) | 259 (12.7) | 1707 (13.4) | 1561 (13.3) | 146 (13.9) | 262 (11.5) | 495 (11.6) |
| 2 | 20,674 (19.2) | 303 (18.6) | 91 (16.9) | 360 (17.6) | 2016 (15.8) | 1839 (15.7) | 177 (16.9) | 353 (15.4) | 702 (16.5) |
| 3 | 20,961 (19.5) | 296 (18.2) | 103 (19.1) | 381 (18.6) | 2349 (18.4) | 2154 (18.4) | 195 (18.6) | 443 (19.4) | 811 (19.1) |
| 4 | 22,934 (21.3) | 346 (21.3) | 115 (21.4) | 446 (21.8) | 3030 (23.8) | 2806 (24.0) | 224 (21.4) | 531 (23.2) | 942 (22.2) |
| 5 (most deprived) | 23,662 (22.0) | 460 (28.3) | 149 (27.7) | 593 (29) | 3624 (28.4) | 3320 (28.4) | 304 (29.0) | 694 (30.4) | 1298 (30.5) |
| Unknown | 432 (0.4) | 4 (0.2) | 1 (0.2) | 4 (0.2) | 22 (0.2) | 20 (0.2) | 2 (0.2) | 2 (0.1) | 4 (0.1) |
| Townsend score, mean [SD] | 0.28 [3.17] | 0.90 (3.3) | 0.93 (3.4) | 0.96 (3.2) | 0.88 (3.2) | 0.87 [3.2] | 0.92 [3.3] | 1.06 (3.23) | 1.05 (3.2) |
| Townsend fifth, mean [SD] | 3.11 [1.41] | 3.33 (1.4) | 3.31 (1.4) | 3.37 (1.4) | 3.38 (1.4) | 3.38 [1.4] | 3.34 [1.4] | 3.46 (1.36) | 3.43 (1.3) |
| **Mean time on database at LMP (years)** | 7.62 [4.94] | 8.18 (5.3) | 8.21 (5.3) | 8.08 (5.2) | 9.59 (4.9) | 9.63 [4.9] | 9.29 [5.3] | 8.09 (5.0) | 8.02 (5.1) |
| **Pregnancy end date in** |  |  |  |  |  |  |  |  |  |
| 2000 | 7642 (7.1) | 41 (2.5) | 10 (1.9) | 61 (3) | 413 (3.2) | 396 (3.4) | 17 (1.6) | 91 (4) | 165 (3.9) |
| 2001 | 7915 (7.4) | 55 (3.4) | 11 (2) | 87 (4.3) | 503 (3.9) | 474 (4.1) | 29 (2.8) | 139 (6.1) | 229 (5.4) |
| 2002 | 8174 (7.6) | 89 (5.5) | 23 (4.3) | 119 (5.8) | 563 (4.4) | 523 (4.5) | 40 (3.8) | 146 (6.4) | 261 (6.1) |
| 2003 | 9109 (8.5) | 133 (8.2) | 45 (8.4) | 180 (8.8) | 741 (5.8) | 686 (5.9) | 55 (5.2) | 207 (9.1) | 391 (9.2) |
| 2004 | 9766 (9.1) | 110 (6.8) | 23 (4.3) | 154 (7.5) | 916 (7.2) | 855 (7.3) | 61 (5.8) | 161 (7) | 312 (7.3) |
| 2005 | 10,132 (9.4) | 151 (9.3) | 48 (8.9) | 204 (10) | 1120 (8.8) | 1016 (8.7) | 104 (9.9) | 201 (8.8) | 411 (9.7) |
| 2006 | 10,487 (9.7) | 137 (8.4) | 44 (8.2) | 178 (8.7) | 1271 (10) | 1183 (10.1) | 88 (8.4) | 230 (10.1) | 404 (9.5) |
| 2007 | 10,949 (10.2) | 186 (11.4) | 66 (12.3) | 225 (11) | 1555 (12.2) | 1433 (12.2) | 122 (11.6) | 253 (11.1) | 464 (10.9) |
| 2008 | 11,074 (10.3) | 196 (12.1) | 73 (13.6) | 229 (11.2) | 1707 (13.4) | 1569 (13.4) | 138 (13.2) | 272 (11.9) | 483 (11.4) |
| 2009 | 10,948 (10.2) | 244 (15) | 81 (15.1) | 278 (13.6) | 1875 (14.7) | 1698 (14.5) | 177 (16.9) | 292 (12.8) | 533 (12.5) |
| 2010 | 11,377 (10.6) | 283 (17.4) | 114 (21.2) | 328 (16.1) | 2084 (16.3) | 1867 (16.0) | 217 (20.7) | 293 (12.8) | 599 (14.1) |
| **Body mass index (BMI) nearest before LMP** |  |  |  |  |  |  |  |  |  |
| <20 | 8677 (8.1) | 131 (8.1) | 30 (5.6) | 171 (8.4) | 1251 (9.8) | 1155 (9.9) | 96 (9.2) | 202 (8.8) | 359 (8.4) |
| 20 – 24 | 31,869 (29.6) | 446 (27.4) | 151 (28.1) | 543 (26.6) | 3881 (30.4) | 3577 (30.6) | 304 (29.0) | 633 (27.7) | 1167 (27.4) |
| 25 – 29 | 16,923 (15.7) | 302 (18.6) | 100 (18.6) | 377 (18.5) | 2413 (18.9) | 2204 (18.8) | 209 (19.9) | 406 (17.8) | 761 (17.9) |
| 30 – 34 | 7178 (6.7) | 165 (10.2) | 55 (10.2) | 209 (10.2) | 1196 (9.4) | 1069 (9.1) | 127 (12.1) | 188 (8.2) | 372 (8.7) |
| >34 | 4609 (4.3) | 117 (7.2) | 52 (9.7) | 162 (7.9) | 947 (7.4) | 853 (7.3) | 94 (9.0) | 175 (707) | 336 (7.9) |
| unknown | 38,317 (35.6) | 464 (28.6) | 150 (27.9) | 581 (28.4) | 3060 (24) | 2842 (24.3) | 218 (20.8) | 681 (29.8) | 1257 (29.6) |
| BMI, mean [SD] | 24.83 [5.79] | 25.98 (6.5) | 26.81 (7) | 26.13 (6.6) | 25.62 (6.4) | 25.56 [6.4] | 26.29 [6.8] | 25.87 (6.7) | 25.99 (6.7) |

Notes:

Exclusions: all congenital anomalies, terminations of pregnancy for foetal anomalies (TOPFA), stillbirths, multiple births (twins, triplets and quadruplets [no higher multiples in the dataset]), exposure to insulin, anti-epileptic drugs (AEDs) or coumarins in the quarter preceding pregnancy and the first trimester, heavy drinking/substance misuse (any record).

Some numbers have been blurred to avoid revealing numbers <5 in other cells.

§ numbers in this category <5, and therefore combined with the category above to avoid disclosure of low numbers.

BMI is body mass index, LMP is last menstrual period, t2-t3 represents trimesters 2 and 3, P1_t1 represents the quarter before pregnancy and the first trimester.

Townsend scores and ranks, see note to Table 2a, reference 89

#### Table B. Infants excluded from the analysis. Table B1. Infants exposed to Insulin or Anti-epileptic drugs [AEDs]

|  | exclusions = anomalies + TOPFAs [n=113316] | | | | | exclusions = anomalies + TOPFAs [n=113316] | | | | |
| --- | --- | --- | --- | --- | --- | --- | --- | --- | --- | --- |
|  | insulin in trimester 1 | | | | | AEDs in trimester 1 | | | | |
|  | Exposed n [%] | % without unknown outcome | Unexposed n [%] | % without unknown outcome | unadjusted OR [95% CI | Exposed n [%] | % without unknown outcome | Unexposed n [%] | % without unknown outcome | unadjusted OR [95% CI |
| **Prematurity** 2000-2010 |  |  |  |  |  |  |  |  |  |  |
| <37 weeks' gestation | 231 [33.58] | na | 7863 [6.98] | na | 6.67 [5.88, 7.69] | 112 [9.62] | na | 7982 [7.12] | na | 1.39 [1.14, 1.69] |
| >=37 weeks' gestation | 457 [66.42] | na | 104,765 [93.02] | na | 0.15 [0.13, 0.17] | 1052 [90.38] | na | 104170 [92.88] | na | 0.72 [0.59, 0.88] |
| <37 to 32 weeks' gestation | 200 [29.07] | na | 6491 [5.76] | na |  | 82 [7.04] | na | 6609 [5.89] | na |  |
| <32 weeks' gestation | 31 [4.51] | na | 1372 [1.22] | na | 3.83 [2.66, 5.51] | 30 [2.58] | na | 1373 [1.22] | na | 2.13 [1.48, 3.08] |
| unknown | 0 |  | 0 |  |  | 0 |  | 0 |  |  |
| total | 688 [100] |  | 112,628 [100] |  |  | 1164 [100] |  | 112,152 [100] |  |  |
| **SGA** 2000-2010 |  |  |  |  |  |  |  |  |  |  |
| <10th centile | 23 [3.34] | 3.8 | 9852 [8.75] | 8.94 | 0.4 [0.27, 0.61] | 121 [10.4] | 10.66 | 9754 [8.7] | 8.89 | 1.22 [1.01, 1.47] |
| >=10th centile | 582 [84.59] | 96.2 | 100,356 [89.1] | 91.06 | 2.48 [1.64, 3.77] | 1014 [87.11] | 89.34 | 99,924 [89.1] | 91.11 | 0.82 [0.68, 0.99] |
| <10th to 3rd centile | 19-22 [<5] | 3.64 | 7711-7714 [6.8] | 7 |  | 99 [8.51] | 8.72 | 7637 [6.81] | 6.96 |  |
| <3rd centile | <5 [<5] | 0.17 | 2135-2138 [1.9] | 1.94 |  | 22 [1.89] | 1.94 | 2117 [1.89] | 1.93 | 1.00 [0.65, 1.52] |
| Total without unknowns | 605 [87.94] | 100 | 110,208 [97.85] | 100 |  | 1135 [97.51] | 100 | 109,678 [97.79] | 100 |  |
| unknown | 83 [12.06] |  | 2420 [2.15] |  |  | 29 [2.49] |  | 2474 [2.21] |  |  |
| total | 688 [100] |  | 112,628 [100] |  |  | 1164 [100] |  | 112,152 [100] |  |  |
| **Breastfeeding** 2004-2010 |  |  |  |  |  |  |  |  |  |  |
| **at birth** |  |  |  |  |  |  |  |  |  |  |
| yes | 165 [23.98] | 45.83 | 25,930 [23.02] | 52.3 | 0.77 [0.63, 0.95] | 226 [19.42] | 40.5 | 25,869 [23.07] | 52.38 | 0.62 [0.52, 0.73] |
| no | 195 [28.34] | 54.17 | 23654 [21] | 47.7 |  | 332 [28.52] | 59.5 | 23,517 [20.97] | 47.62 |  |
| Total without unknowns | 360 [52.33] | 100 | 49,584 [44.02] | 100 |  | 558 [47.94] | 100 | 49,386 [44.03] | 100 |  |
| unknown | 328 [47.67] |  | 63,044 [55.98] |  |  | 606 [52.06] |  | 62,766 [55.97] |  |  |
| total | 688 [100] |  | 112,628 [100] |  |  | 1164 [100] |  | 112,152 [100] |  |  |
| **at 6-8 weeks** |  |  |  |  |  |  |  |  |  |  |
| yes | 75 [10.9] | 25 | 13,,102 [11.63] | 32.59 | 0.69 [0.53, 0.90] | 84 [7.22] | 19.31 | 13,093 [11.67] | 32.68 | 0.49 [0.39, 0.63] |
| no | 225 [32.7] | 75 | 27100 [24.06] | 67.41 |  | 351 [30.15] | 80.69 | 26,974 [24.05] | 67.32 |  |
| Total without unknowns | 300 [43.6] | 100 | 40,202 [35.69] | 100 |  | 435 [37.37] | 100 | 40,067 [35.73] | 100 |  |
| unknown | 388 [56.4] |  | 72,426 [64.31] |  |  | 729 [62.63] |  | 72,085 [64.27] |  |  |
| total | 688 [100] |  | 112,628 [100] |  |  | 1164 [100] |  | 112,152 [100] |  |  |

#### Table B2. Infants exposed to coumarins or heavy drinking or substance misuse (exclusions = anomalies + TOPFAs [n=113316])

|  | coumarins [anticoagulants] in trimester 1 | | | | | heavy drinking/ substance misuse | | | | |
| --- | --- | --- | --- | --- | --- | --- | --- | --- | --- | --- |
|  | Exposed n [%] | % without unknown outcomes | Unexposed n [%] | % without unknown outcomes | unadjusted OR [95% CI | Exposed n [%] | % without unknown outcomes | Unexposed n [%] | % without unknown outcomes | unadjusted OR [95% CI |
| **Prematurity** 2000-2010 |  |  |  |  |  |  |  |  |  |  |
| <37 weeks' gestation | 60 [15.71] | na | 8034 [7.11] | na | 2.44 [1.85, 3.23] | 198 [11.03] | na | 7896 [7.08] | na | 1.61 [1.41, 1.89] |
| >=37 weeks' gestation | 322 [84.29] | na | 104,900 [92.89] | na | 0.41 [0.31, 0.54] | 1597 [88.97] | na | 103,625 [92.92] | na | 0.62 [0.53, 0.71] |
| <37 to 32 weeks' gestation | 48 [12.57] | na | 6643 [5.88] | na |  | 163 [9.08] | na | 6528 [5.85] | na |  |
| <32 weeks' gestation | 12 [3.14] | na | 1391 [1.23] | na | 2.60 [1.46, 4.63] | 35 [1.95] | na | 1368 [1.23] | na | 1.60 [1.14, 2.25] |
| unknown | 0 |  | 0 |  |  | 0 |  | 0 |  |  |
| total | 382 [100] |  | 112,934 [100] |  |  | 1795 [100] |  | 111,521 [100] |  |  |
| **SGA** 2000-2010 |  |  |  |  |  |  |  |  |  |  |
| <10th centile | 42 [10.99] | 11.17 | 9833 [8.71] | 8.9 | 1.28 [0.93, 1.79] | 294 [16.38] | 17.03 | 9581 [8.59] | 8.78 | 2.13 [1.89, 2.44] |
| >=10th centile | 334 [87.43] | 88.83 | 100,604 [89.08] | 91.1 | 0.78 [0.56, 1.07] | 1432 [79.78] | 82.97 | 99,506 [89.23] | 91.22 | 0.47 [0.41, 0.53] |
| <10th to 3rd centile | 28 [7.33] | 7.45 | 7708 [6.83] | 6.98 |  | 222 [12.37] | 12.86 | 7514 [6.74] | 6.89 |  |
| <3rd centile | 14 [3.66] | 3.72 | 2125 [1.88] | 1.92 | 1.97 [1.15, 3.37] | 72 [4.01] | 4.17 | 2067 [1.85] | 1.89 | 2.25 [1.77, 2.87] |
| Total without unknowns | 376 [98.43] | 100 | 110,437 [97.79] | 100 |  | 1726 [96.16] | 100 | 109,087 [97.82] | 100 |  |
| unknown | 6 [1.57] |  | 2497 [2.21] |  |  | 69 [3.84] |  | 2434 [2.18] |  |  |
| total | 382 [100] |  | 112,934 [100] |  |  | 1795 [100] |  | 111,521 [100] |  |  |
| **Breastfeeding** 2004-2010 |  |  |  |  |  |  |  |  |  |  |
| at birth |  |  |  |  |  |  |  |  |  |  |
| yes | 57 [14.92] | 39.58 | 26,038 [23.06] | 52.29 | 0.60 [0.43, 0.84] | 294 [16.38] | 35.94 | 25,801 [23.14] | 52.52 | 0.51 [0.44, 0.59] |
| no | 87 [22.77] | 60.42 | 23762 [21.04] | 47.71 |  | 524 [29.19] | 64.06 | 23,325 [20.92] | 47.48 |  |
| Total without unknowns | 144 [37.7] | 100 | 49,800 [44.1] | 100 |  | 818 [45.57] | 100 | 49,126 [44.05] | 100 |  |
| unknown | 238 [62.3] |  | 63,134 [55.9] |  |  | 977 [54.43] |  | 62,395 [55.95] |  |  |
| total | 382 [100] |  | 112,934 [100] |  |  | 1795 [100] |  | 111,521 [100] |  |  |
| at 6-8 weeks |  |  |  |  |  |  |  |  |  |  |
| yes | 16 [4.19] | 14.68 | 13161 [11.65] | 32.58 | 0.36 [0.21, 0.61] | 117 [6.52] | 17.94 | 13,060 [11.71] | 32.77 | 0.45 [0.37, 0.55] |
| no | 93 [24.35] | 85.32 | 27,232 [24.11] | 67.42 |  | 535 [29.81] | 82.06 | 26,790 [24.02] | 67.23 |  |
| Total without unknowns | 109 [28.53] | 100 | 40,393 [35.77] | 100 |  | 652 [36.32] | 100 | 39,850 [35.73] | 100 |  |
| unknown | 273 [71.47] |  | 72,541 [64.23] |  |  | 1143 [63.68] |  | 71,671 [64.27] |  |  |
| Total | 382 [100] |  | 112,934 [100] |  |  | 1795 [100] |  | 111,521 [100] |  |  |

***Table B3. Infants excluded from the analysis: those with congenital anomalies or multiples***

|  | exclusions = anomalies + TOPFAs [n=113316] | | | | | no exclusions [n=117717] | | | | |
| --- | --- | --- | --- | --- | --- | --- | --- | --- | --- | --- |
|  | multiples | | | | | anomalies including TOPFAs | | | | |
|  | Exposed n [%] | % without unknown outcomes | Unexposed n [%] | % without unknown outcomes | unadjusted OR [95% CI | Exposed n [%] | % without unknown outcomes | Unexposed n [%] | % without unknown outcomes | unadjusted OR [95% CI |
| **Prematurity** 2000-2010 |  |  |  |  |  |  |  |  |  |  |
| <37 weeks' gestation | 1326 [51.22] | na | 6768 [6.11] | na | 16.67 [14.29, 16.67] | 1219 [27.7] | na | 8094 [7.14] | na | 16.67 [14.29, 16.67] |
| >=37 weeks' gestation | 1263 [48.78] | na | 103,959 [93.89] | na | 0.06 [0.06, 0.07] | 3182 [72.3] | na | 105,222 [92.86] | na | 0.06 [0.06, 0.07] |
| <37 to 32 weeks' gestation | 1042 [40.25] | na | 5649 [5.1] | na |  | 478 [10.86] | na | 6691 [5.9] | na |  |
| <32 weeks' gestation | 284 [10.97] | na | 1119 [1.01] | na | 12.07 [10.53, 13.84] | 741 [16.84] | na | 1403 [1.24] | na | 16.13 [14.71, 17.86] |
| unknown | 0 |  | 0 |  |  | 0 |  | 0 |  |  |
| total | 2589 [100] |  | 110727 [100] |  |  | 4401 [100] |  | 113316 [100] |  |  |
| **SGA** 2000-2010 |  |  |  |  |  |  |  |  |  |  |
| <10th centile | 255 [9.85] | 10.14 | 9620 [8.69] | 8.88 | 1.16 [1.01, 1.32] | 489 [11.11] | 13.39 | 9875 [8.71] | 8.91 | 1.16 [1.01, 1.32] |
| >=10th centile | 2259 [87.25] | 89.86 | 98,679 [89.12] | 91.12 | 0.86 [0.76, 0.99] | 3162 [71.85] | 86.61 | 100,938 [89.08] | 91.09 | 0.86 [0.76, 0.99] |
| <10th to 3rd centile | 198 [7.65] | 7.88 | 7538 [6.81] | 6.96 |  | 348 [7.91] | 9.53 | 7736 [6.83] | 6.98 |  |
| <3rd centile | 57 [2.2] | 2.27 | 2082 [1.88] | 1.92 | 0.85 [0.65, 1.10] | 141 [3.2] | 3.86 | 2139 [1.89] | 1.93 | 2.04 [1.72, 2.43] |
| Total without unknowns | 2514 [97.1] | 100 | 108,299 [97.81] | 100 |  | 3651 [82.96] | 100 | 110,813 [97.79] | 100 |  |
| unknown | 75 [2.9] |  | 2428 [2.19] |  |  | 750 [17.04] |  | 2503 [2.21] |  |  |
| total | 2589 [100] |  | 110,727 [100] |  |  | 4401 [100] |  | 113,316 [100] |  |  |
| **Breastfeeding** 2004-2010 |  |  |  |  |  |  |  |  |  |  |
| at birth |  |  |  |  |  |  |  |  |  |  |
| yes | 487 [18.81] | 52.14 | 25,608 [23.13] | 52.25 | 1.00 [0.88, 1.13] | 700 [15.91] | 47.78 | 26,095 [23.03] | 52.25 | 1.00 [0.88, 1.13] |
| no | 447 [17.27] | 47.86 | 23,402 [21.13] | 47.75 |  | 765 [17.38] | 52.22 | 23,849 [21.05] | 47.75 |  |
| Total without unknowns | 934 [36.08] | 100 | 49,010 [44.26] | 100 |  | 1465 [33.29] | 100 | 49,944 [44.07] | 100 |  |
| unknown | 1655 [63.92] |  | 61,717 [55.74] |  |  | 2936 [66.71] |  | 63,372 [55.93] |  |  |
| total | 2589 [100] |  | 110,727 [100] |  |  | 4401 [100] |  | 113,316 [100] |  |  |
| at 6-8 weeks |  |  |  |  |  |  |  |  |  |  |
| yes | 196 [7.57] | 25.42 | 12,981 [11.72] | 32.67 | 0.70 [0.60, 0.83] | 315 [7.16] | 26.72 | 13,177 [11.63] | 32.53 | 0.70 [0.60, 0.83] |
| no | 575 [22.21] | 74.58 | 26,750 [24.16] | 67.33 |  | 864 [19.63] | 73.28 | 27,325 [24.11] | 67.47 |  |
| Total without unknowns | 771 [29.78] | 100 | 39,731 [35.88] | 100 |  | 1179 [26.79] | 100 | 40,502 [35.74] | 100 |  |
| Unknown | 1818 [70.22] |  | 70,996 [64.12] |  |  | 3222 [73.21] |  | 72,814 [64.26] |  |  |
| total | 2589 [100] |  | 11,0727 [100] |  |  | 4401 [100] |  | 113,316 [100] |  |  |

#### Table C. Premature birth and exposure to SSRI [any] in trimesters 2 or 3 [n=107573]

|  | **Exposed n [%]** | **Unexposed [n] [%]** | **totals** | **unadjusted OR [95% CI]** | **adjusted* OR [95% CI]** | **Other significant variables in the model** | **adjusted* OR [95% CI]** |
| --- | --- | --- | --- | --- | --- | --- | --- |
| ≥37 weeks' gestation | 1501 [92.37] | 99,915 [94.31] | 101,416 | 1.37[1.14-1.65] | 1.19[0.97-1.46] | Parity [Primiparous] | 1.11[1 .05-1.18] |
| <37 weeks' gestation | 124 [7.63] | 6033 [5.69] | 6157 |  |  | Townsend_Fifth[1]**  2nd most affluent | 1.2[1.10-1.31] |
| Total | 1625 [100] | 105,948 [100] | 107,573 |  |  | Townsend_Fifth[2]  Middle | 1.27[1.16-1.38] |
|  |  |  |  |  |  | Townsend_Fifth[3]  2nd most deprived | 1.11[1.02-1.21] |
|  |  |  |  |  |  | Townsend_Fifth[4] most deprived | 1.19[1.09-1.29] |
|  |  |  |  |  |  | Smoking | 1.27[1.20-1.34] |
|  |  |  |  |  |  | Depression | 1.26[1.16-1.36] |
|  |  |  |  |  |  |  |  |
| ≥32 weeks' gestation | 1605 [98.77] | 105,035 [99.14] | 106,640 | 1.43 [0.92-2.24] | 1.24[0.76-2.03] | Parity [Primiparous] | 1.44[1.25-1.66] |
| <32 weeks' gestation | 20 [1.23] | 913 [0.86] | 933 |  |  | Townsend_Fifth**[4]  Most deprived | 1.29[1.03-1.62] |
| total | 1625 [100] | 105,948 [100] | 107,573 |  |  | Smoking | 1.38[1.19-1.59] |
|  |  |  |  |  |  | Depression | 1.33[1.09-1.62] |

0 infants had unknown gestation

Exclusions and definitions as in Table 2a

* adjusted for Parity, SES [Townsend fifth], Year of birth [YOB], Smoking, Depression

**compared with the least deprived

Interaction terms ‘Depression by SSRI in trimesters 2 or 3: <32 weeks 1.04[0.39-2.81]

<37 weeks 1.03[0.68-1.54]

#### Table D Small for gestational age and exposure to SSRI [any] in trimesters 2 or 3 [n=105,331]

|  | **Exposed n [%]** | **Unexposed n [%]** | **totals** | **unadjusted OR [95% CI]** | **adjusted** OR [95% CI]** | **Other significant variables in the model** | | **adjusted* OR [95% CI]** | |  |
| --- | --- | --- | --- | --- | --- | --- | --- | --- | --- | --- |
| ≥10th centile | 1421 [89.54] | 94,753 [91.33] | 96,174 |  |  | Primiparous | | 1.70[1.63-1.79] | |  |
| <10th centile | 166 [10.46] | 8991 [8.67] | 9157 | 1.231[1.047-1.448] | 1.09[0.91-1.30] | Townsend_Fifth[1]** | | 1.37[1.26-1.48] | |  |
|  |  |  |  |  |  | Townsend_Fifth[2] | | 1.35[1.25-1.45] | |  |
|  |  |  |  |  |  | Townsend_Fifth[3] | | 1.25[1.17-1.34] | |  |
| total with data | 1587 [100] | 103,744 [100] | 105,331 |  |  | Townsend_Fifth[4] | | 1.18[1.11-1.26] | |  |
| Missing | 38 | 2204 | 2242 |  |  | Smoking | | 2.17[2.07-2.27] | |  |
| Total | 1625 [100] | 105,948 [100] | 107,573 |  |  |  | |  | |  |
|  |  |  |  |  |  |  | |  | |  |
| ≥3rd centile | 1538 [96.91] | 101,826 [98.15] | 103,364 | 1.691[1.268-2.256] | 1.457[1.061-2] | Primiparous | | 1.91[1.73-2.10] | |  |
|  |  |  |  |  |  | Townsend_Fifth[1] | | 1.40[1.19-1.65] | |  |
| <3rd centile | 49 [3.09] | 1918 [1.85] | 1967 |  |  | Townsend_Fifth[2] | | 1.27[1.09-1.47] | |  |
|  |  |  |  |  |  | Townsend_Fifth[3] | | 1.29[1.11-1.49] | |  |
| Total with data | 1587 [100] | 103,744 [100] | 105,331 |  |  | Smoking | | 2.53[2.29-2.80] | |  |
| Unknown | 38 | 2204 | 2242 |  |  |  | |  | |  |
| Total | 1625 | 105,948 | 107,573 |  |  |  | |  | |  |
| Interaction terms ‘Depression by SSRI in trimesters 2 or 3: <10th centile 0.88[0.69-1.13]  <3rd centile 1.11[0.59-2.11]  2242 infants could not be allocated to a birth weight centile. | | | | | | |  | |  | |
|  | | | | | | | | | | |
| * adjusted for Parity, SES [Townsend fifth], Year of birth [YOB], Smoking, Depression. Depression was not a significant variable. | | | | | | | | | | |

** compared with the least deprived .

Exclusions and definitions as in Table 2a

#### Table E1 Breastfeeding [any] at 6-8 weeks – full models [n=38,725]

|  | | | | | |  |  |  |
| --- | --- | --- | --- | --- | --- | --- | --- | --- |
| **SSRI [any] t2 or t3** | **exposed n [%]** | **unexposed [n [%]** | **unadjusted OR [95% CI** | **adjusted** OR [95% CI]** | **Other significant variables in model** | | **aOR [95% CI]*** |  |
| *Yes* | 137 [21.24] | 12,656 [33.24] | 0.54 [0.45-0.66] | 0.77[0.62-0.95] | PARITY[Primiparous] | | 1.06[1.01-1.11] |  |
| No | 508 [78.76] | 25,424 [66.76] |  |  | Townsend_Fifth[1]** | | 0.82[0.76-0.89] |  |
| Total | 645 [100] | 38,080 [100] |  |  | Townsend_Fifth[2] | | 0.70[0.65-0.75] |  |
| Missing data | 980 | 67,868 |  |  | Townsend_Fifth[3] | | 0.59[0.55-0.64] |  |
| Total | 1625 | 105,948 |  |  | Townsend_Fifth[4] | | 0.47[0.43-0.51] |  |
|  |  |  |  |  | Smoking | | 0.43[0.40-0.45] |  |
|  |  |  |  |  | Depression Recorded | | 0.77[0.72-0.83] |  |
| SSRI high dose t2 or t3 |  |  |  |  |  | |  |  |
| Yes | 47 [21.96] | 12,746 [33.1] | 0.57 [0.41-0.79] | 0.45[0.23-0.86] | Parity [primiparous] | | 1.06[1.01-1.11] |  |
| No | 167 [78.04] | 25,765 [66.9] |  |  | Townsend_Fifth[1]** | | 0.82[0.76-0.89] |  |
| Total | 214 [100] | 38,511 [100] |  |  | Townsend_Fifth[2] | | 0.70[0.65-0.75] |  |
| Missing data | 324 | 68,524 |  |  | Townsend_Fifth[3] | | 0.59[0.55-0.64] |  |
| Total | 538 | 107,035 |  |  | Townsend_Fifth[4] | | 0.47[0.43-0.51] |  |
|  |  |  |  |  | Smoking | | 0.43[0.40-0.45] |  |
|  |  |  |  |  | Depression | | 0.76[0.70-0.82] |  |
|  |  |  |  |  |  | |  |  |
| Interaction terms ‘Depression by SSRI in trimesters 2 or 3: any dose 1.11[0.72-1.70]  High dose 2.49[1.13-5.52], in final model  * adjusted for Parity, SES [Townsend fifth], Year of birth [YOB], Smoking, Depression. Depression was not a significant variable. | | | | | | | | |

** compared with the least deprived .

Exclusions and definitions as in Table 2a

#### Table E2 Breastfeeding at birth – full models [n=47726]

| **SSRI in trimesters 2 or 3** | **Exposed n [%]** | **Unexposed [n [%]** | **unadjusted OR [95% CI** | **adjusted** OR [95% CI]** | **Other Significant Variables In Model** | **aOR [95% CI]*** |  |
| --- | --- | --- | --- | --- | --- | --- | --- |
| Yes | 306 [37.09] | 24,819 [52.92] | 0.53 [0.46-0.61] | 0.66[0.56-0.77] | Yob[2008] | 1.20[1.07-1.34] |  |
| No | 519 [62.91] | 22,082 [47.08] |  |  | Yob[2009] | 1.18[1.05-1.32] |  |
| total | 825 [100] | 46,901 [100] |  |  | Yob[2010] | 1.14[1.01-1.28] |  |
| unknown | 800 | 59,047 |  |  | Multiparty | 0.84[0.80-0.87] |  |
| total | 1625 | 105,948 |  |  | Townsend_Fifth[1]** | 0.84[0.78-0.90] |  |
|  |  |  |  |  | Townsend_Fifth[2] | 0.64[0.59-0.68] |  |
|  |  |  |  |  | Townsend_Fifth[3] | 0.50[0.47-0.54] |  |
|  |  |  |  |  | Townsend_Fifth[4] | 0.36[0.34-0.39] |  |
|  |  |  |  |  | Smoking | 0.46[0.44-0.48] |  |
|  |  |  |  |  | Depression | 0.87[0.82-0.92] |  |
|  | |  |  |  |  |  |  |
| **High dose SSRI in trimesters 2 or 3** |  |  |  |  |  |  |  |
| Yes | 101 [36.07] | 25,024 [52.74] | 0.51 [0.40-0.65] | 0.59[0.44-0.78] | Yob[2008] | 1.20[1.07-1.34] |  |
| No | 179 [63.93] | 22,422 [47.26] |  |  | Yob[2010] | 1.14[1.01-1.28] |  |
| total | 280 [100] | 47,446 [100] |  |  | Multiparty | 0.84[0.80-0.87] |  |
| unknown | 258 | 59,589 |  |  | Townsend_Fifth[1]** | 0.84[0.78-0.90] |  |
| total | 538 | 107,035 |  |  | Townsend_Fifth[2] | 0.64[0.59-0.68] |  |
|  |  |  |  |  | Townsend_Fifth[3] | 0.50[0.47-0.54] |  |
|  |  |  |  |  | Townsend_Fifth[4] | 0.36[0.34-0.39] |  |
|  |  |  |  |  | Smoking | 0.46[0.44-0.48] |  |
|  |  |  |  |  | Depression | 0.86[0.81-0.91] |  |
| Interaction terms ‘Depression by SSRI in trimesters 2 or 3: any dose 1.24[0.89-1.74]  High dose 1.65[0.90-3.01]  * adjusted for Parity, SES [Townsend fifth], Year of birth [YOB], Smoking, Depression. Depression was not a significant variable. | | | | | | | |

** compared with the least deprived

Exclusions and definitions as in Table 2a
