# Supplementary material for: Antidepressant prescriptions, discontinuation, depression and perinatal outcomes, including breastfeeding: A population cohort analysis
Source: PLoS One. 2019 Nov 18;14(11):e0225133. doi: 10.1371/journal.pone.0225133 (PMC6860440; doi:10.1371/journal.pone.0225133)
Supplement: Attachment — Submitted filename: Rebuttal letter PONE.docx [file pone.0225133.s004.docx]

Rebuttal letter PONE-D-19-17104

3.10.19

Dear Professor Hashimoto,

Thank you for the return of our manuscript. We are very grateful for the reviewers’ support and suggestions. We have done our best to comply.

We hope that readers will concur with reviewer 2: I enjoyed reading this paper. This is a valuable paper that presents evidence of the associations between antidepressant prescriptions, discontinuation, depression and perinatal outcomes, including breastfeeding.

Thank you for your time spent on this paper.

Yours,

Sue Jordan, on behalf of all authors

| Reviewer #1: |  |
| --- | --- |
| Dear authors, This manuscript presents data based on a population cohort to study associations between exposure to antidepressants, their discontinuation, depression and preterm birth, small for gestational and breastfeeding at 6-8 weeks. The data presented are incredibly valuable and valid, however I would recommend to explain and demonstrate why these results are important? | Thank you. We have added to lines 446-452:  The concentration of prescribing and adverse outcomes amongst the economically disadvantaged should encourage pre-conception monitoring *in tandem* with review of antidepressants and non-pharmacological therapies. These data indicate that prescription of antidepressants is an **important marker** for adverse outcomes, easily identified in primary care records. This could, and should, be used to trigger additional monitoring, including third trimester scans or alternative continuous monitoring technology to detect SGA. *Using prescriptions to target care before, during and after pregnancy* |
| How they can contribute to the practice? what can be done regarding depression prevention? | We agree, it is essential to explain how the findings contribute to practice. We hope that the implications for practice derived from the study are set out clearly above. Prevention of depression is an important area, but outside the scope of the data presented. |
| The manuscript lacks information, reflection and soundness. In addition, it seems that authors were just describing results (because data was available) and were not doing it for a specific propose. This feeling begins in introduction. | Thank you: we have addressed this. The information from the study is contained in 9 tables, 5 with 8 multivariate models. To ensure the paper is of a manageable length, readers are referred to earlier work for methodological details at points (references 33 and 17). We have added to the statement of purpose / aim, line 63 *et seq:*  … the key question for women and clinicians - the harm to benefit balance of starting, stopping or continuing antidepressant pharmacotherapy [5] - remains unanswered. Accordingly, the aim of this study is to investigate …  This is a retrospective analysis of prospectively collected routine health service data. Our data shares many of the limitations of similar cohort studies. One of our unique features is that we hold data on breastfeeding at 6-8 weeks and prescribed medicines. |
| A more reflective way on implications for practice would improve your manuscript. | The implications for practice are summarised in the ‘Conclusion and Implications’ section, to which we have added, lines 455-464, above. We feel that some caution is needed before drawing causal inferences from observation data, and our main implication for practice is the need to target groups identified for additional care. We have added, line 437:  Drawing causal inferences from observation data may be imprudent. |
| Please explain better how depression was measured, by whom? What manual/diagnosis system they used?  can you guarantee they use the same methods?  This need more detail since it is the main outcome. | Thank you, to clarify, we have added, line 109 *et seq*:  Depression was defined as any diagnosis of depression in the woman’s record before the end of trimester 1 [91 days after 1st day of LMP], recorded by the GP using Read codes, version 2 [17, 36, 37]  We acknowledge the limitations of using the diagnoses of GPs, lines 400 *et seq*.  We acknowledge the risks of under-ascertainment or inconsistency between individual GPs, and the limitations of taking the absence of records as ‘no problems’.  We agree that more detail will improve the value of the paper. Therefore, we have added the S1 file, to give full details of the codes GPs used to record depression. |
| Reviewer #2: |  |
| The authors investigated the associations between exposure to antidepressants, their discontinuation, depression and preterm birth, small for gestational age (SGA), breastfeeding at 6-8 weeks. They found that exclusive formula feeding at 6-8 weeks was associated with prescriptions in trimesters 2 or 3 for any antidepressants, SSRIs, particularly higher doses, discontinuation of antidepressants or SSRIs after trimester 1, diagnosis of depression, particularly if medicated, rather than unmedicated. Moreover, preterm birth at <37 and <32 weeks’ gestation was associated with diagnosis of depression, particularly if medicated; birth at <37 weeks was associated with antidepressants, SGA <3rd centile was associated with antidepressants, and SSRIs, particularly higher doses. They stated that both depression and antidepressants indicated increased risks of exclusive formula feeding at 6-8 weeks. | Thank you |
| These findings will be of interest to practitioners, as well as researchers in the field. | Thank you |
| I have the following concerns.  1.Abstract Line 59. Conclusions. What do the authors conclude from the results that intra-uterine exposure to antidepressants and maternal depression were associated with adverse perinatal outcomes? If possible, the authors should comment on the results. | Thank you. Our comment has been modified, and the section retitled ‘implications’:  **Implications:** Exposure to antidepressants or depression increased risks of exclusive formula feeding at 6-8 weeks, and prescription of antidepressants was associated with SGA <3^rd^ centile. Prescription of antidepressants offers a **useful marker** to target additional support and additional care before and during pregnancy and lactation.  We feel that some caution is needed before drawing causal inferences from observation data, and our main implication for practice is the need to target groups identified for additional care. This is now stated under ‘limitations’. We have added, line 446:  Drawing causal inferences from observation data may be imprudent. |
| 2. The amount of the presented tables is very large, and this is confusing for readers. This aspect of the paper should be improved. Table 2a, 2b,and 2c show the infants excluded from the analysis. If possible, Table 2 could be transferred to the section of supplementary tables. | This is very helpful. Done. |
| 3. Discussion. Line 275. “To our knowledge, this is the first report that antidepressants, particularly high dose SSRIs, depression, particularly medicated depression, and discontinuation of antidepressants adversely affect breastfeeding rates at 6-8 weeks; the effects of high doses and discontinuation were not independent of depression.” The authors claim that this is the first report. But what do they mean by “first report”? | Thank you. We have clarified by adding ‘in the published literature’. Line 272:  To our knowledge, this is the first report, in the published literature, |
| Line 423, The authors also stated that “In Wales, the relationships between economic deprivation and breastfeeding, [55, 56] and SSRI prescription in pregnancy, adjusted for diagnosed depression are linear. [48]. This statement indicates that other researchers have also reported the relationships between breastfeeding, [55, 56] and SSRI prescription in pregnancy. The authors should clarify the new findings they found. | Thank you, we have clarified, lines 354-5.  In Wales, linear relationships between economic deprivation and breastfeeding, [58, 59] and economic deprivation and SSRI prescription in pregnancy, adjusted for diagnosed depression, have been identified. [50]  These studies were authored by our group. |
| In conclusion, I enjoyed reading this paper. This is a valuable paper that presents evidence of the associations between antidepressant prescriptions, discontinuation, depression and perinatal outcomes, including breastfeeding. | Thank you. We hope that others will concur, and modify practice to help prevent some of these adverse outcomes in future. |
| Please ensure that your manuscript meets PLOS ONE's style requirements, including those for file naming. | Files have been renamed e.g. Tables A1, A2 in S2 file.  Headings are black, without italics  Tables have been moved alongside the text  The limitations have been moved from the start to the end of the discussion. The reference numbers were changed accordingly.  Fonts have been checked.  (These changes were not tracked to preserve readability.)  The data availability and full details of the funding were removed from the manuscript. |
| Email of 15^th^ October from Emma Thompson  1..We note your current Data Availability Statement:  "All relevant data are within the manuscript and its Supporting Information files. Additional data are available in the EUROmediCAT report and its appendices. No patient level data are available under the terms of ethical and governance reviews. No participant consent is obtained for population level studies. Individual records for all databases were anonymised, and individual patient data cannot be publicly deposited or fully shared upon request. They are only available directly from the database providers. The corresponding author will endeavour to meet requests for further data. All other interested parties are able to apply to obtain the data in the same way as the current investigators"  Before we proceed with your submission, kindly address the following:  A) Please fully disclose the methods of data collection from database providers in the interest of researchers to replicate or verify the findings of your study, or indicate if you have already done so in your manuscript (i.e. Methods).  B) Please provide any relevant data set names, variables, accession codes, URLs, DOIs, etc. that an independent researcher would need in order to request your minimal data set from the database providers.  C) PLOS ONE's data policy requires a non-author institutional contact for data access in the interest of maintaining long-term data accessibility. At this time, please provide a non-author point of contact that is able to field data access queries (i.e. email address of database providers. | Our statement now reads:  All relevant data are within the manuscript and its Supporting Information files.  Additional data are available in the EUROmediCAT report and its appendices (reference 33 - publically available).  The datasets generated and analysed during the current study are not publicly available, because the anonymised data, held by SAIL, can only be accessed within the SAIL secure remote access environment within the context of an approved project, following governance review.  No patient level data are available under the terms of ethical and governance reviews. No participant consent is obtained for population level studies. Individual records for all databases were anonymised, and individual patient data cannot be publicly deposited or fully shared upon request. They are only available directly from the database providers. All other interested parties are able to apply to obtain the data in the same way as the current investigators. Data used in the study can be accessed within the SAIL secure environment subject to approval by the SAIL Information Governance Review Panel. The URL for the SAIL databank is: https://saildatabank.com/, and the non-author contacts are Cynthia McNerney, Information Governance Manager, The SAIL Team, Swansea University: [c.l.mcnerney@swansea.ac.uk](mailto:c.l.mcnerney@swansea.ac.uk) and Charlotte Arkley, Administrator, SAIL Databank [c.r.arkley@swansea.ac.uk](mailto:c.r.arkley@swansea.ac.uk).  Dataset information (names of datasets and provenance) is provided under 'Methods, subsection Setting'. As indicated in the text, further details are in reference 33.  The corresponding author will endeavour to meet requests for further data and information. |
| Email of 22.10.10  Before we proceed, please confirm whether any of the data that are not publicly available (EUROmediCAT reprot and its appendices, data sets accessed within SAIL) are part of the minimal data set. | We had added:  We confirm that none of the data that are not publicly available is part of the minimal data set. |
